# Conditions underpinning success in joint service-education workforce planning

**DOI:** 10.1186/1478-4491-7-17

**Published:** 2009-02-25

**Authors:** Mary Ellen Purkis, Barbara Herringer, Lynn Stevenson, Laureen Styles, Jocelyne Van Neste-Kenny

**Affiliations:** 1 Faculty of Human & Social Development, University of Victoria, Victoria, BC, Canada; 2 Health & Human Services, Camosun College, Victoria, BC, Canada; 3 Professional Practice & Nursing, Vancouver Island Health Authority, Victoria, BC, Canada; 4 Health & Human Services, Vancouver Island University, Nanaimo, BC, Canada; 5 Health & Human Services, North Island College, Campbell River, BC, Canada

## Abstract

Vancouver Island lies just off the southwest coast of Canada. Separated from the large urban area of Greater Vancouver (estimated population 2.17 million) by the Georgia Strait, this geographical location poses unique challenges in delivering health care to a mixed urban, rural and remote population of approximately 730 000 people living on the main island and the surrounding Gulf Islands. These challenges are offset by opportunities for the Vancouver Island Health Authority (VIHA) to collaborate with four publicly funded post-secondary institutions in planning and implementing responses to existing and emerging health care workforce needs.

In this commentary, we outline strategies we have found successful in aligning health education and training with local health needs in ways that demonstrate socially accountable outcomes. Challenges encountered through this process (i.e. regulatory reform, post-secondary policy reform, impacts of an ageing population, impact of private, for-profit educational institutions) have placed demands on us to establish and build on open and collaborative working relationships. Some of our successes can be attributed to evidence-informed decision-making. Other successes result from less tangible but no less important factors. We argue that both rational and "accidental" factors are significant – and that strategic use of "accidental" features may prove most significant in our efforts to ensure the delivery of high-quality health care to our communities.

## Introduction

Delivery of health services in Canada has been under significant stress for at least the past decade [1]. The reason most often cited as causing this stress is the limited number of professional and paraprofessional workers available to staff the admittedly well-resourced system characteristic of this country. Canada's health care system (particularly hospital and physician services) operates almost solely on the basis of a system of public finance [2]. A significant guiding principle of the Canadian health care system is that citizens will experience timely access to insured health services on a prepaid basis, without direct charges at the point of service. Demographic forecasting identifies a future of particular importance to those involved in ensuring the viability of this public health care system: a reduction in numbers of students entering the secondary and post-secondary education system – that is, potential future health care workers – and a concurrent ever-increasing number of older adults. Indeed, Canada leads the developed world in projected increase in population over the age of 65 by the year 2030 [3].

Such demographic forecasting has encouraged us to seek ways to work together to solve our mutual concerns: for the post-secondary sector this involves a strong and steady supply of well-qualified applicants for programmes; for the health sector the concern revolves around having a steady supply of appropriately qualified applicants to fill vacant staff positions. This collective work has been largely positive, but it is worth noting that an educator's idea of an excellent graduate (that is, one who engages critically with ideas and can write about those ideas in a clear and concise way) does not always match an employer's idea of an ideal employee (that is, one who will fit into the work setting quickly and effectively).

We have encountered some significant challenges, and it is our view that an explication of the challenges may have wider utility than just our own jurisdiction. In this paper, we present first a brief description of the scope of our work together. We move on to outline the strategies we have used and tools that we have available to us to support this work. We conclude with some of the key issues we have encountered in our work. As far as possible, we have drawn on available evidence to inform our work. But we have also seized opportunities available to us perhaps more by accident rather than through purely rational processes. We believe these accidental factors have had a significant role in our success and therefore hold them as important to acknowledge and document.

### Demographic challenges framing the scope for planning work

The demographic pattern we face on the Island is similar to that faced by every health region across Canada, and no doubt in many other western societies – that of shrinking child and youth populations with growing older adult populations [3]. This pattern suggests three combined challenges for anyone engaging in the work of planning for health human resources: first, a reduction in the number of citizens available to enrol in health programmes at the same time that we will be facing the second challenge of record numbers of retirements out the health worker ranks, and finally, an increasing aged population requiring care. In addition, one of the "accidents" of our particular situation is that Vancouver Island, located just off Canada's most southwesterly point, has for many years been recognized as a retirement haven. The moderate climate and spectacular natural beauty of the island draws many Canadians in their post-retirement years. So, on top of the general demographic trend, on this Island of nearly three-quarters of a million people, we have an added challenge related to the complex demands associated with older adult care.

People living on the Island obtain health care through the Vancouver Island Health Authority . Of all the professionals working within the Health Authority, the largest population educated on the Island is registered nurses. There are four public, post-secondary institutions on the Island: two in the provincial capital of Victoria (Camosun College and University of Victoria), one located mid-Island (Vancouver Island University – formerly Malaspina University College) and one at the north end of the Island (North Island College). All these institutions offer education to prepare registered nurses (RN) with an entry-level credential of a baccalaureate degree (four-year programme). Several also offer licensed practical nurse (LPN) programmes (one-year programme) and a variety of home support and mental health worker programmes. Education for physicians has recently become possible on the Island through a collaborative arrangement with the province's one medical programme, located in Vancouver. Allied health professional programming (e.g. physiotherapy, occupational therapy, nutrition, radiation therapy, respiratory therapy) has historically been offered only in Vancouver, although practical experience is often gained in health facilities operated by VIHA.

## Discussion

Over the past three years, senior leaders from the Vancouver Island Health Authority have been meeting with the academic deans from the four Island post-secondary institutions. These face-to-face meetings have been extremely helpful in assisting the educators to better understand the critical and emergent needs for a variety of health professionals/paraprofessionals and workers, the specific knowledge and skills most needed within the organization, and the mapping of a complete inventory of the educational preparation for health professionals available on the Island. Initial meetings created many positive opportunities to recognize that some of the Health Authority's most pressing needs could be addressed either through existing programming or through the creative re-development of purpose-built programmes. While the numbers of staff requiring educational support in technically specific fields was often very small, we discovered that we could meet these needs with minimal financial investment from the educational programmes because the necessary competences could be learned from existing programming. Examples here are workshops related to mental health challenges in older adults, education workshops for clinical educators, and so on.

Beyond these solutions to immediate issues and needs, the cross-jurisdictional meetings between service providers and educators have prompted discussions about the viability of developing new programming in the areas of specialty nursing preparation; education for physical, occupational, respiratory, radiation therapists; and a range of imaging technicians to provide long-term sustainability in the allied health fields for the Health Authority. It is necessary for us to balance those concerns for long-term sustainability with the cost and viability of developing such programmes.

Funding for public, post-secondary institutions comes primarily from the provincial government. Government representatives monitor enrolments very carefully. Post-secondary institutions that are unable to maintain anticipated student enrolment levels are frequently disadvantaged in subsequent year funding allocations, and so these decisions must be thought through very carefully.

One of the "accidents" of our collaborative work has been a deepening of our understanding of one another's contexts for public funding. What might previously have been interpreted as a lack of willingness to collaborate on solutions is now understood as a lack of capacity to respond within given resource constraints. Emerging from such recognitions, we have identified a consistent process as to how particular requests for formal education programmes come to the Island Deans to ensure that there has been sufficient exploration of current and future human resource needs within VIHA and that the request is based on available evidence. This process has had the effect of reducing the amount of effort put into developing and offering technically specific courses for very few individuals. Post-secondary educators are also now able to consider how they can supplement the efforts of clinical educators to meet organizationally specific needs.

### Strategies and resources that support our work

Our collective planning work has evolved from primarily information sharing to more strategic discussions about how institutions can support the mid-range expectations of human resources in VIHA, based on assessments of population health. Face-to-face meetings have been essential to move forward. Such meetings are possible for us by virtue of the ease of travel from one end of the Island to the other. We have excellent transportation infrastructure and those who travel are reimbursed for their travel costs by their institutions. Face-to-face meetings have enabled us to develop strong working relationships. Our relationships have enabled other local problems (e.g. issues related to student practice placements, uneven policy implementation in clinical settings, etc.) to be solved in a respectful manner.

In addition, the four Deans had long-standing relationships arising out of a pre-existing collaboration for nursing education [4] and the four institutions are involved in specific partnering relationships. Support also comes in the form of a Memorandum of Understanding (MOU) between Camosun College, North Island College and Vancouver Island University signed by the institutional presidents that supports principles of collaboration and joint activities whenever possible. The deans at these three institutions then moved this institutional MOU into a more local agreement specific to health and human services education.

We have also been able to tap into organizational re-design work that is occurring at the same time as our planning work is evolving. So, for instance, VIHA is developing a human resource planning document at the senior executive level and educators have been invited to take part in the process of the development of that plan. This represents a key opportunity to ensure that the plan that is developed makes the best use of local educator resources – and also aids in our considerations of which new programmes should take highest priority within our respective institutions.

Although we are seeking to keep our minds open to the full range of health human resource need, we are all conscious that nursing represents the largest population of health care workers in our system. Nursing human resource planning therefore presents one of the most significant challenges in terms of supply and retention. The Health Authority is undertaking a major re-design project entitled Care Delivery Model Re-design or CDMR. This project is intended to assist the Health Authority to develop new care delivery models that reflect the Health Authority's responsibilities to deliver health care to the Island's population within the current context of significant shortage of all health professionals, but primarily nurses [5]. A representative from the educator group has been invited to take part in this planning process, and this cross-jurisdictional collaboration is intended to support our future planning for educational programming.

### Key challenges to planning for sustainable health human resources

Challenges abound as we move through our collaborative work together. It is certainly the case that the time and effort we have invested in building our relationships together has helped us maintain our commitment to work through our current challenges together. Nonetheless, issues persist in making this work more difficult than it is often portrayed in the literature on this topic. Some of the issues we face are as follows.

#### Introduction of private, for-profit educational providers

Under the banner of increasing choice for students, the provincial Ministry of Advanced Education has had an explicit goal of expanding opportunities for private education institutions to open access for students within the Province [6]. While representing a diverse sector with varied histories and practices, reports indicate that some of these institutions engage in "aggressively marketing programmes to students, regardless of the suitability of the programme to the student's needs or abilities [7]. Remaining separate from the collaborative planning exercises described above, these businesses have the potential to "flood the market" with students, often having a negative knock-on effect in the clinical practice field. The negative effect of private education providers is not purely ideological and demands further explication.

In part as a response to the challenge of being able to hire sufficient numbers of staff as well as changing locations of care (e.g. moving long-term care patients into community facilities), the overall effect of health care restructuring through the 1990s has been to reduce the size of the inpatient sector, a clinical setting where group placements have been used to maximize faculty supervision of pre-registration students. Models of practice education have not kept pace with these service sector changes and, as a result, we experience overcrowding of students and increased expressions of exhaustion on the part of staff working on inpatient units. The actual impact on patient care is rarely recorded nor acknowledged, but we anticipate that it, too, is likely not positive.

The forms of accountability to communities and government are also very different for these two types of educational providers. Public post-secondary institutions operate within a relatively transparent context, with much of the programme information, including success rates, accessible to public scrutiny. By contrast, private educational institutions have no specific requirement to provide accountability to the community – only profits for stakeholders. This means that once the immediate need to train a specified number of health care workers has been satisfied, the programme may cease to be offered and often the educational institution disappears, if it was ever physically located in the community in the first place. Where the community-based public institution was experiencing difficulties recruiting qualified students and offering them relevant practice experiences as part of their programme, they may have diverted their limited public funding to other programming areas – only to be accused of not responding to community needs once the private educational provider has ceased operations.

#### Different planning timeframes

Educational institutions operate largely on the basis of the annual academic calendar. Holidays for faculty are often scheduled for July and August, and students are often given a break from schoolwork at the same time – although many students use this time to work in order to help to fund their studies. In September, everyone is rested and a great deal of energy goes into developing programmes of orientation that invite students back to class and practice experiences and the cycle begins over again.

Within the practice world, the cycle revolves around annual budget cycles, with numerous mid-course corrections possible. New programmes can begin – or end – at any time that patients' needs demand. Staff members take vacations around the year at times that respond, as much as possible, to ensuring that staffing needs on any given unit are met. In rural and remote areas, services often close down entirely because of staff vacations. This can present challenges in relation to the provision of a full range of necessary practice placements for students.

These represent quite different planning contexts. As needs for including new learning opportunities for students into a curriculum arise, it can take a minimum of a year to ensure that change is made, evaluated and implemented into the formal curriculum design for any given health programme. Where the care context changes and new care providers are needed (a recent example might be the development of legislation for nurse practitioners in the province), a period longer than a year may be required in order that not only the curriculum can be developed but that qualified faculty can be hired, regulations approved by provincial regulatory bodies and qualifying examinations developed by regulators and passed by students.

While provincial government representatives and health authority personnel occupy a world characterized by rapid and substantial change, the slower pace of the public, post-secondary sector can result in frustration and feeling that one groups' challenges are not being addressed by the other group with as much urgency as the other feels they should. These challenges are mirrored in the fact that educators in our system hold line accountability to the Ministry of Advanced Education and Labour Market Development (AELMD), while those in the health authorities are responsible for reporting to the Ministry of Health Services (MOHS). Collaborative relationships, including instituting formal liaison positions, are created only where ministers and deputy ministers work on the basis of strong, collaborative relationships. This again represents a site of contingency: it has been our experience that when, seemingly by accident, we work within a context of collaboration between these two significant ministries, our work and our success are greatly enhanced. When there is not a spirit of collaboration present between the ministries, our efforts become significantly deflected when we have to address priorities of one that do not match priorities of the other. A recent example here has been a policy decision within the Ministry of Health Services to support a particular curricular model for RN education (a three-year, "condensed" programme) that distracts from the Ministry of Advanced Education's wider policy context to increase the number of RNs educated, regardless of curriculum model.

#### Regulatory bodies

The province of British Columbia currently regulates 24 different health professions under umbrella legislation called the Health Professions Act. Each regulated profession has developed a College of professional members and public members appointed by the provincial government. These professional colleges are given their mandate and powers through the Health Professions Act. The regulation for each profession sets out reserved titles, scope of practice for registrants and reserved actions.

Regulatory bodies are designed to protect the public and, when they operate at their very best, ensure careful, informed, third party oversight on any changes in the scope of practice of any given professional group. The presence of regulatory bodies ensures that the educational preparation for practice is appropriate and relevant for the job description established by any given employer and that employers cannot ask practitioners with insufficient education to undertake tasks for which they have not received training.

Things do not always work as smoothly in practice and, under conditions where employers are seeking flexibility in the workforce, either in relation to entry qualifications (as is often seen when they seek to hire foreign health professionals) or when professional groups seek to expand their scope of practice, necessitating longer educational programmes (as was seen when registered nurses sought to have the baccalaureate degree become the required entry-level qualification for professional practice), regulatory bodies are often caught in between these pressing demands. As in our example above of the different planning timeframes, where curriculum changes are extensive and may require review by a regulatory body, time to institute needed changes extends and is often perceived by employers as placing unacceptable constraints on their mandate to provide timely care for members of the community they serve.

### Immediacy of workforce needs during a period of significant organizational change

This challenge is an age-old tension for both educators and employers. Sitting just outside the day-to-day demands of providing health care for an ageing population, educators can take the long view as they contemplate curriculum changes. Educators take the task of preparing professionals for a practice world seriously and, while seeking to make continuing education an achievable option for all graduates, also know that often the entry-level education experience will, for many practitioners, be the final formal education they will receive. Additional education for these staff will, in all likelihood, come in the form of short staff development opportunities designed by employers to meet their wider system demands.

Under these circumstances, the interest for educators becomes not just to prepare practitioners for today's world of health care but to ensure that graduates look further to think about and make positive and active contributions to a valued form of health care practice into the future. For instance, many nursing curricula are founded on the principles of primary health care [8]. These principles can be used by nursing personnel to raise questions about the validity of current practices when compared with evidence-informed expectations based on population health outcome goals [9]. But the value of a new graduate seeking to address population health needs when his more experienced RN peers are expecting a new staff member who will accept and fulfil his or her share of the overall unit workload may well result in significant conflict and concerns being raised as to the relevance of the new graduate's preparation for the contemporary workplace.

As the need for nursing personnel increases and where service delivery is disrupted due to the inability of a health authority to hire sufficient numbers of nurses, an inevitable desired response is to shorten nursing programmes, focus the curricula increasingly on skill development for acute care practice and dismiss the principles of primary health care as irrelevant in the current context. Recognizing the source of such interpretations and the validity of the criticism within the context from which it arises, educators can but continue to seek opportunities to engage in dialogue with senior health authority leaders in relation to the short-sightedness of a focus on skills in the absence of measures that demonstrate the long-term value of population-based health care.

## Conclusion

On the basis of the work we do together across the jurisdictional boundaries of post-secondary education and the provision of primary, secondary and tertiary health care, our relationships have been a critically important factor in enabling us to make significant strides towards an integrated approach to health human resource planning. It may seem trite to draw attention to the idea of relationships. It is a word that, perhaps especially in health care, tends towards overuse. But in this particular circumstance, while in our individual and collective planning we rely on published evidence to help us understand the implications of our collective work, it has been our relationships that have helped us take the best advantage of the opportunities that arise.

The previously established network of educators has come to the work of planning with a single health authority with knowledge and trust that, even where provincial policy shifts introduce increased propensity towards competition, we can continue to address the needs of the health authority as a unified group of educators. We are working in a world where such relationships have been tested to the breaking point in the past [4] and so we know that we need to take best advantage from our current mutual interests in working collaboratively.

So, while we rely on published evidence to design our educational curricula and to plan for changes in care delivery models, the context we are working in does not always produce anticipated results even when we work in relation to established policy guidelines. Instead, what we document in this paper is the contingent reality of the world of health care policy and practice that we operate in. The relationships we describe are unique to our circumstances – yet we expect that our efforts at making forward progress on the seemingly intractable issues related to health human resource planning will likely sound very familiar to our readers.

The legislative context of the Canadian health care system is markedly different from that of our neighbours to the south, while being somewhat similar to the system in the United Kingdom and Australia. Yet the significance of our ability to take advantage of the "accidental" and contingent opportunities provided to us by living on an Island that enables us to meet regularly and respond to education/practice issues within the relative coherence of four post-secondary institutions responding to the needs of just one health authority cannot be dismissed. Just 30 kilometers across the Straits of Georgia, in the metropolitan area of Vancouver, there are at least seven public, post-secondary institutions seeking to engage in a similar form of planning with three different health authorities, each with its own demands and needs. The opportunities for working at cross-purposes and working for the interests of the most powerful against the interests of the less powerful – instead of for the benefit of the collective – are many and often counterproductive to building and maintaining relationships.

In many ways, our Island location protects us from some of the difficulties of contemporary health human resource planning – but not from all those that arise out of deeper structural issues such as the neoliberal agenda related to privatizing education and health care that places unrealistic economic pressures on public institutions and that encourages a focus on short-term solutions over longer-range problem identification and collective solution generation. For these we must continue to take advantage of our positive working relationships to exert pressure and commit to our mutual engagement in a critical dialogue that helps to bring these structural relations into view and to plan in light of them – rather than in their shadows.

## Abbreviations

AELMD: Ministry of Advanced Education and Labour Market Development, Province of British Columbia; CDMR: Care Delivery Model Redesign; MOH: Ministry of Health; Province of British Columbia; MOU: Memorandum of Understanding; VIHA: Vancouver Island Health Authority

## Competing interests

The authors declare that they have no competing interests.

## Authors' contributions

All authors contributed to the collegial dialogue regarding health human resource planning that is described in this paper. MEP wrote the initial draft of the paper. All authors reviewed the paper and made suggestions on revising the final manuscript. All approved the final manuscript.
